# Supplementary figures and images for: Tumor-Associated Neutrophils Are a Negative Prognostic Factor in Early Luminal Breast Cancers Lacking Immunosuppressive Macrophage Recruitment
Source: Cancers (Basel). 2024 Sep 15;16(18):3160. doi: 10.3390/cancers16183160 (PMC11430230; doi:10.3390/cancers16183160)

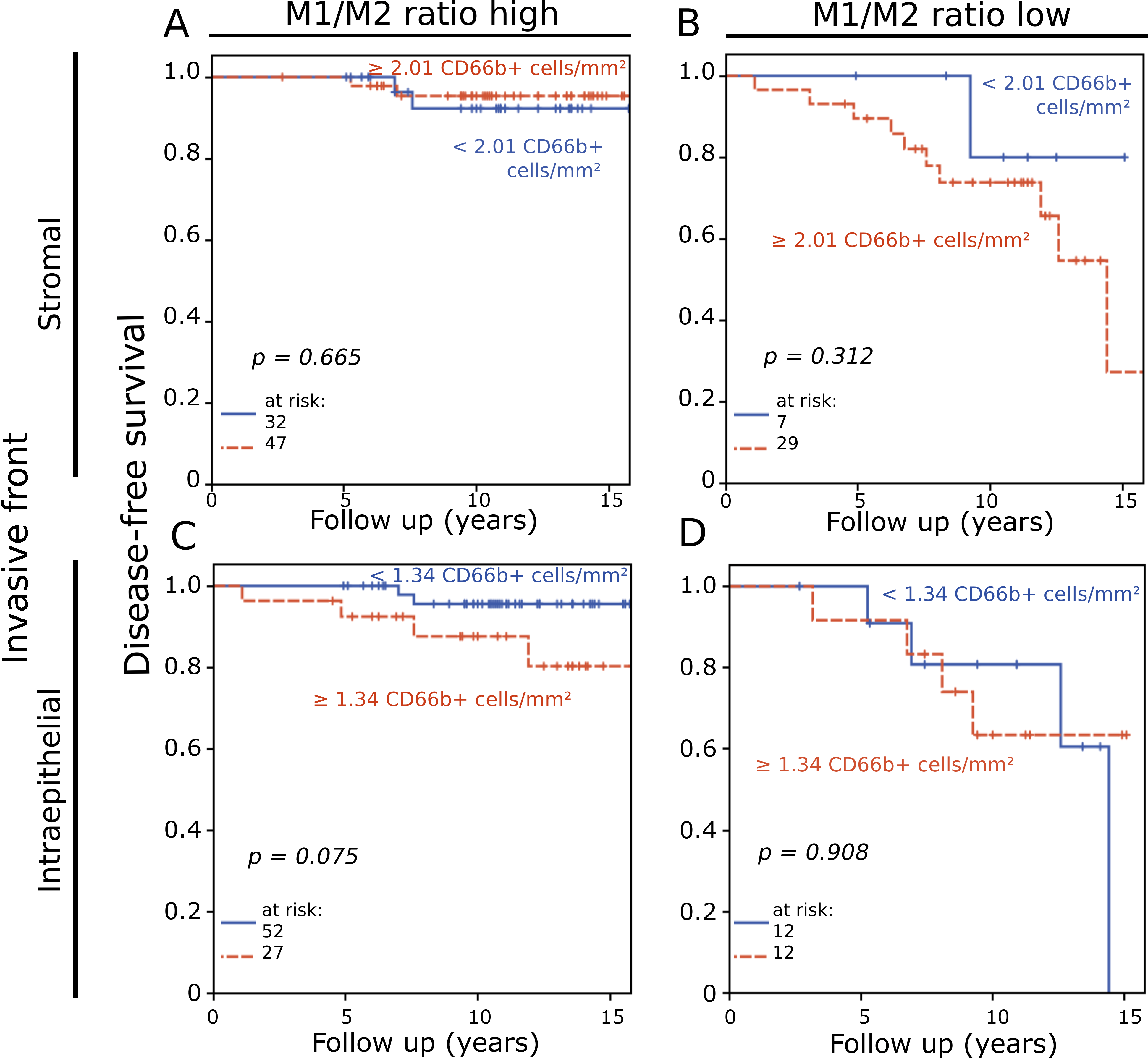

Supplement: Supplementary file 1 [file cancers-16-03160-s001.zip › SupplementaryFigure1.tiff]
